# Supplementary material for: Manipulating and visualizing the dynamic aggregation-induced emission within a confined quartz nanopore
Source: Nat Commun. 2018 Sep 7;9:3657. doi: 10.1038/s41467-018-05832-y (PMC6128826; doi:10.1038/s41467-018-05832-y)
Supplement: Supplementary file 1 — Supplementary Information [file 41467_2018_5832_MOESM1_ESM.pdf]

Supplementary Information for

Manipulating and Visualizing the Dynamic Aggregation-Induced-Emission Confining within a Quartz Nanopore

Ying et al.

Supplementary Methods

Chemicals

All the reagents were analytical grades, acetonitrile(AR), potassium chloride(AR), were purchased from Sigma-Aldrich Co., Ltd., USA. tetra-n-butylammonium hexafluorophosphate, were purchased from J&K Scientific Ltd, China. The ultrapure water (18.2 MΩ cm at 25 ℃) were made from Milli-Q system, USA. The AIE molecule (DMTPS-DCV) were synthetized according to the previous researches.[1, 2] The sliver wire (0.5 mm, Alfa Aesar Co., U.S.A.) was used as the electrodes.

Cell culture

MCF-7 cells were cultured in Rosewell Park Memorial Institute (RPMI) 1640 medium (Gibco, Grand Island, New York, USA) supplemented with 10% heat-inactivated bovine serum, penicillin (100 U mL^{-1}) and streptomycin (100 U mL^{-1}). Cells were incubated at 37 °C in a humidified atmosphere of 95% air and 5% CO_2. Cells were washed three times with 10 mM PBS (pH 7.0) buffer before electrochemical measurements.

Fabrication of the quartz nanopore

The quartz nanopore were made from quartz capillaries with 1 mm outer diameter and 0.7 mm inner diameter (QF100-70-7.5, Sutter Instrument Co., Novato, USA). First, the capillaries were cleaned by sonication in acetone, ethanol and deionized water respectively at 60 ℃ for 30 min to remove the organic residual. Then the quartz capillaries were dried by N_2 and drawn down to nanopore with P-2000 laser puller (Sutter Instrument Co., Novato, USA). Heat = 685, Fil = 4, Vel = 45, Del = 170, Pul = 205. The quartz nanopore was characterized using Zeiss Ultra Plus scanning electron microscope (Carl Zeiss, Oberkochen, Germany)

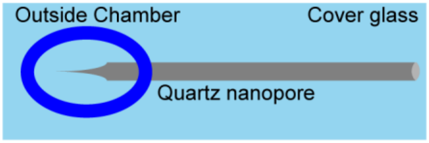

Supplementary Figure 1. The designed cover glass with an outside chamber for filling with the DMTPS-DCV acetonitrile solution. The outside chamber is sealed with the acrylic ab adhesive.

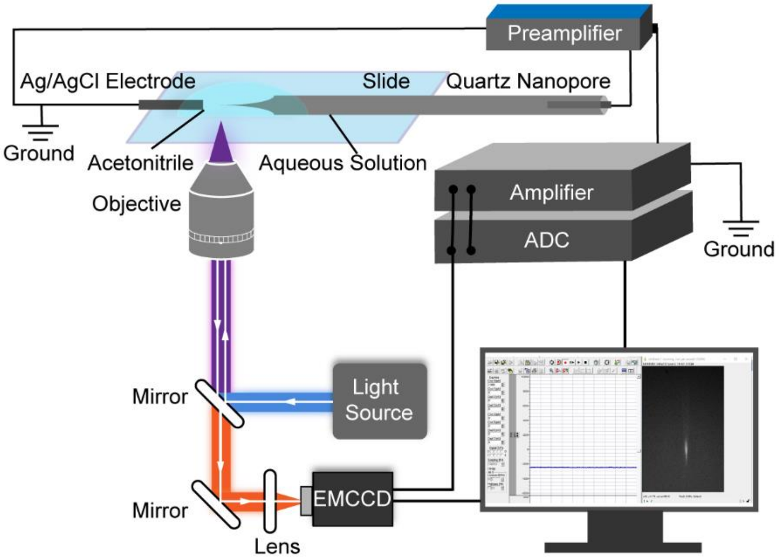

Supplementary Figure 2. Schematic configuration for integrating the inverted fluorescence microscopy with the ultralow ionic current acquisition system. The synchronized collection of electrical and optical signals was guaranteed by the multichannel design of the analog to digital converter (ADC), then monitored by the computer.

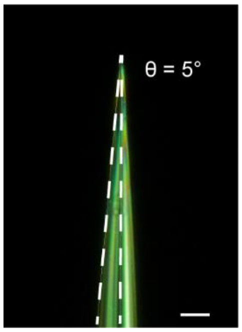

Supplementary Figure 3. The dark-field image of the quartz nanopore. The half-cone angle of the conical-shaped area (θ) is calculated as 5°. Scale bars, 10 μm.

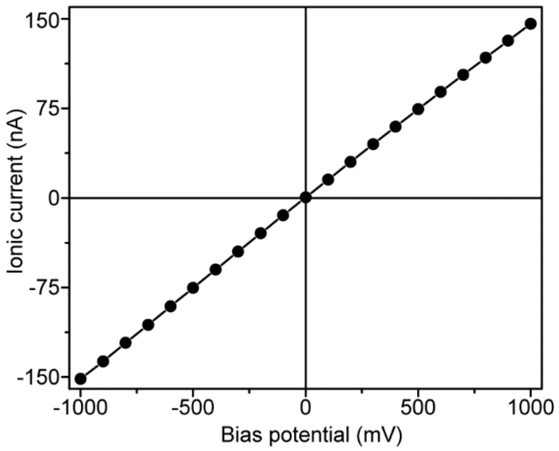

Supplementary Figure 4. The I-V curve of the quartz nanopore at 1 M KCl.

Supplementary Note 1

To describe the potential distribution along the nanopore at room temperature of 298 K, we carried out the finite element method simulation (FEM) by coupled Poisson-Nernst−Planck (PNP) and Navier−Stokes (NS) equations. The COMSOL Multiphysics software (COMSOL Inc., Burlington, MA, USA) is used for the FEM simulation. The geometry of the model nanopore is based on the SEM image of the nanopore which are d = 100 nm, Θ = 5°, $l_{nanopore}$ = 25 μm (Figure 1a and Supplementary Figure 1-2). The surface charge density of the nanopore was set as -10 mC m^{-2}. To accelerate the calculation, the yellow line of boundary ⑤ is defined as surface of glass with surface charge density of -10 mC m^{-2} while green line of boundary ④ set as surface of glass without surface charge density. The 2D axisymmetric geometry of a nanopore for the simulation is shown in Supplementary Figure 5. Note that these geometries are not drawn to scale.

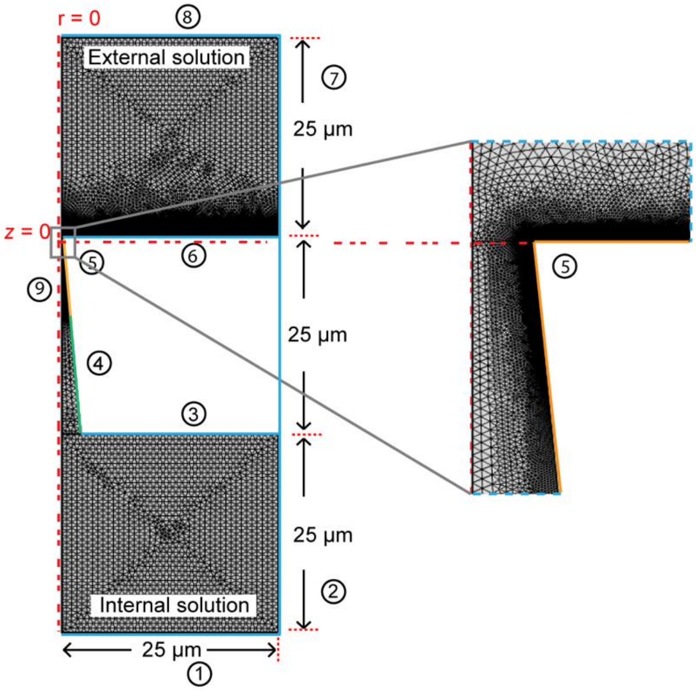

Supplementary Figure 5. The 2D axisymmetric geometry, the mesh for the finite-element simulation, and the boundary settings of the nanopore for the simulation of the potential distribution. Blue lines (①, ②, ③, ⑥, ⑦, ⑧) are the internal bulk solution and external bulk solution. Green line of ④ is the surface of glass. Yellow line of ⑤ is the surface charge of glass.

In order to simplifier the simulation, here, both the outside and inside solution are set as 10 mM KCl. The dynamics of the ions in the channel is governed by the Poisson-Nernst-Planck (PNP) equations which relate surface charge with ionic fluxes and corresponding conductivity distribution within a glass nanopore. The ionic flow is computed by the Nernst−Planck (NP) equation (eq. 1) where the diffusion, migration, and convection terms are included.

$$\boldsymbol{J}_i = -D_i \nabla c_i - \frac{F z_i}{RT} D_i c_i \nabla \Phi + c_i \boldsymbol{u} \qquad (1)$$

In eq. 1, $\boldsymbol{J}_i$ is the ionic flow vector, F is the Faraday's constant, T is the absolute temperature, Φ is the potential, $\boldsymbol{u}$ is the position-dependent fluid velocity, respectively. D_i, C_i, and Z_i represent the diffusion coefficient, the concentration, and the charge of species i in solution, respectively.

As neither species are transported into or out of the surface of glass (e.g. boundaries ②, ③, ④, ⑤, ⑥and ⑦ in 2D axial symmetric model, Supplementary Figure 5), there is no normal flux; the boundary condition is set as:

$N \cdot \boldsymbol{J}_i = 0$

The relationship between the electric potential and ion concentration is described by Poisson equation, eq. 2.

$$\nabla^2 \Phi = -\frac{F}{\epsilon} \sum_i z_i c_i \qquad (2)$$

Where ϵ is the dielectric constant of the medium.

The flow distribution is given by the Navier-Stokes equation, eq. 3, describing the pressure and electrical force driven flow.

$$\boldsymbol{u} \nabla \boldsymbol{u} = \frac{1}{\rho} \left(-\nabla p + \eta \nabla^2 \boldsymbol{u} - F \left(\sum_i \sigma_i c_i\right) \nabla \Phi\right) \quad (3)$$

Here, ρ and η are the density and viscosity of the fluid, and p is the pressure. p sets as 1×10^{-3} kg m^{-3} and η sets as 1×10^{-3} Pa· s.

Numerical solution of the equations involves discretization, which uses a mesh. Representative meshes for the finite-element simulations are shown in Supplementary Figure 5. In each model, right part shows the entirety of the domain and right part illustrates the region at the tip of nanopore. The finite element simulations were carried out with COMSOL Multiphysics 5.2a (COMSOL Inc., Burlington, MA, USA) operated on a Lenovo P500 workstation (Intel(R) Xeon(R) CPU E5-1620 v3@3.50GHz, 4core, 32GB RAM)

Therefore, the simulation results in Supplementary Figure 6 shows the electrical potential distribution along the centerline axis of a 100 nm diameter nanopore with a surface charge -10 mC m^{-2} for applied biases potential at -1 V and 1 V. The FEM demonstrates that the largest fraction of the bias potential drops at the narrowest tip of nanopore with approximately region of 15 μm in length.

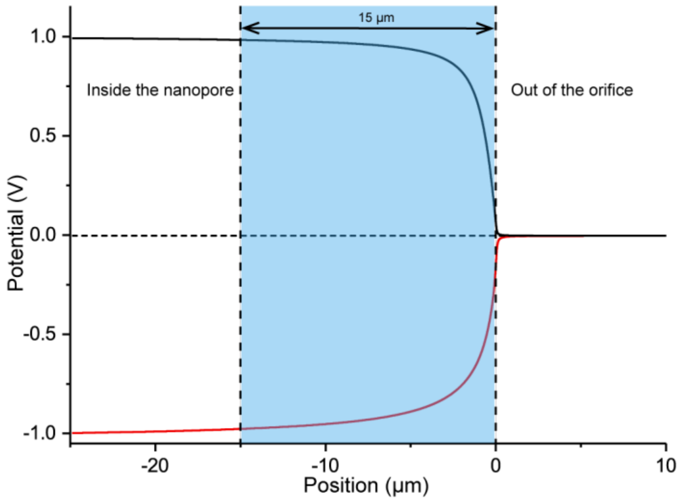

Supplementary Figure 6. Electric potential along the center-line z axis (r = 0) of a 100 nm diameter nanopore in 10 mM KCl as a function of applied bias (ΔV) across the nanopore at −1 V and +1 V, respectively.

Supplementary Note 2

We calculated the number of DMTPS-DCV molecules according to volume of acetonitrile filled inside the nanopore (V_{tip}). According to the Supplementary Figure 3 and 7, the tip volume of nanopore could be considered as a cylinder. Therefore, V_{tip} was calculated as follows

$$V_{tip} = \frac{\pi r^2 h}{3} \qquad (4)$$

Where h is the height of the acetonitrile inside nanopore, r is radius of acetonitrile part. Here, h is 15 μm, r is 1.49 μm which is calculated by $r = \tan\theta \times h$, where θ is the half-cone angle of the conical-shaped area. Therefore, the V_{tip} is 26 fL. Since the concentration of DMTPS-DCV in the outside acetonitrile solution is 10 μM, we assume that 26 fL of 10 μM DMTPS-DCV was driven into the tip of nanopore. Therefore, the number of DMTPS-DCV molecules in the tip of nanopore is estimated to approximately 161000.

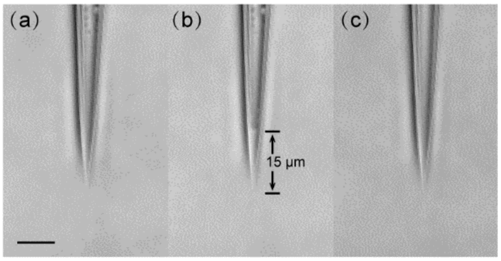

Supplementary Figure 7. Electrochemically ingress of acetonitrile into the aqueous filled quartz nanopore. (a) Initial immersion at 0 V; (b) Ingress of 26 fL acetonitrile after potential was stepped to 1 V; (c) Complete egress of acetonitrile at -1V. The 10 mM TBAPF6 and 10 mM KCl were used as organic and aqueous electrolyte to conduct the ionic flow. Scale bars, 10 μm.

Supplementary Note 3

As the aqueous electrolyte concentration down to 1 mM (Supplementary Figure 8), the applied voltage drops within the bulk solution phase rather than across the liquid/liquid interface. Therefore, the it is hard to achieve the electrochemically controlling the surface tension and the related fluidic motion. As the electrolyte concentration increases to 100 mM (Supplementary Figure 9), the significant decreasing of the nanopore resistance hinders the electrochemical control of the fluid motion. Therefore, 10 mM KCl were used here as electrolyte in inside aqueous solution to efficiently ingress of the acetonitrile solution.

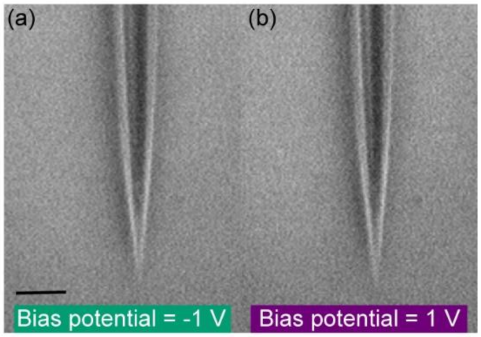

Supplementary Figure 8. Electrochemically ingress of acetonitrile into the aqueous filled quartz nanopore at the KCl concentration of 1 mM. (a) Initial immersion at -1 V; (b) The bias potential was stepped to 1 V. Scale bars, 10 μm. The liquid flow is largely determined by the surface tension effects. The concentration of aqueous electrolyte will affect the liquid flow of AIEgen as described in previous study[3].

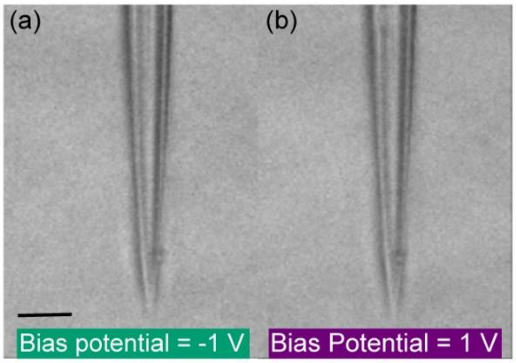

Supplementary Figure 9. Electrochemically ingress of acetonitrile into the aqueous filled quartz nanopore at the KCl concentration of 100 mM. (a) Initial immersion at -1 V; (b) The bias potential was stepped to 1 V. Scale bars, 10 μm.

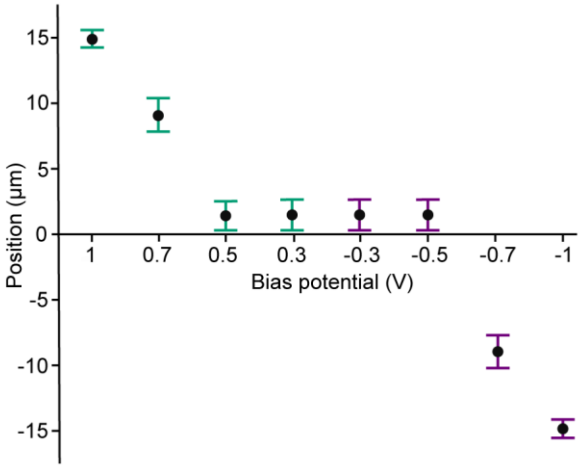

Supplementary Figure 10. Dependence of the bias potential on the electrochemically ingress of acetonitrile into the aqueous filled quartz nanopore at the KCl concentration of 10 mM. The tip of the nanopore defines as z position of 0 μm. Therefore, the ingress of the acetonitrile produces the positive z position value while the egress of the acetonitrile generates the negative z position value. Since ±1 V triggers the acetonitrile moving deepest inside the nanopore, we set the bias potential as ±1 V in the following AIE manipulations. Data are mean ± s.e.m. of three technical replicates.

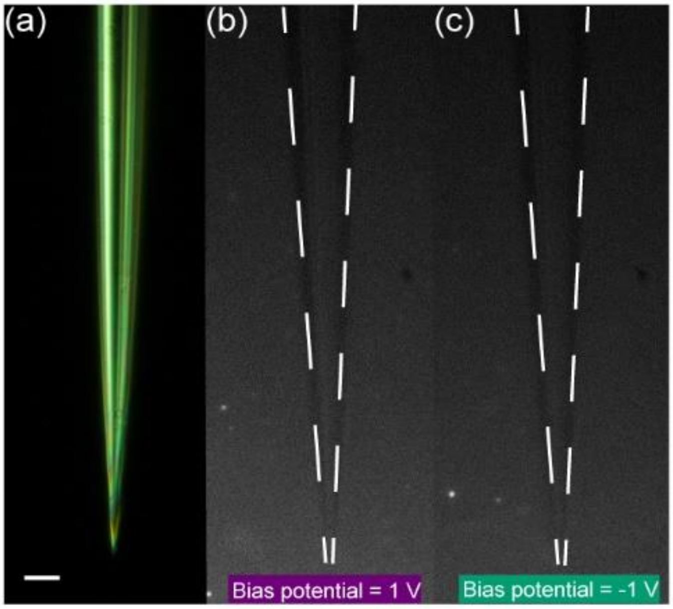

Supplementary Figure 11. The optical images for filling the inside and outside of quartz nanopore with aqueous solution and 10 μM DMTPS-DCV aqueous solution, respectively. (a) Darkfield image at 0V. (b) Fluorescence image at 1 V. (c) Fluorescence image at -1 V. No bright spot was observed inside nanopore cavity. The bright spot in the outside solution indicates the AIE effect of DMTPS-DCV in aqueous solution. Scale bars, 10 μm.

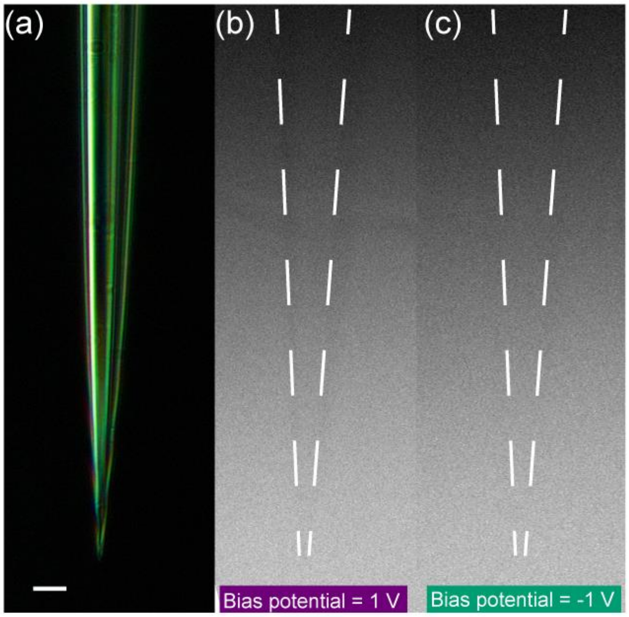

Supplementary Figure 12. The optical images for filling the inside and outside of quartz nanopore with acetonitrile solution and 10 μM DMTPS-DCV acetonitrile solution, respectively. (a) Dark-field image at 0 V. (b) Fluorescence image at 1 V. (c) Fluorescence image at -1 V. No bright spot was observed inside nanopore cavity. Scale bars, 10 μm.

Supplementary Table 1. The *Z* Position of the brightest spot in Figure 2a. The velocity of the movement is calculated based on the movement during the recording time interval of 15 s.

Bias Potential (V)	*Z* Position of the brightest spot (μm)	Recording time (s)	Velocity of the Movement (μm s^{-1})
-1	31	0	N.A.
-1	10	15	1.4
1	43	30	2.2
-1	15	45	1.8
1	46	60	2.1

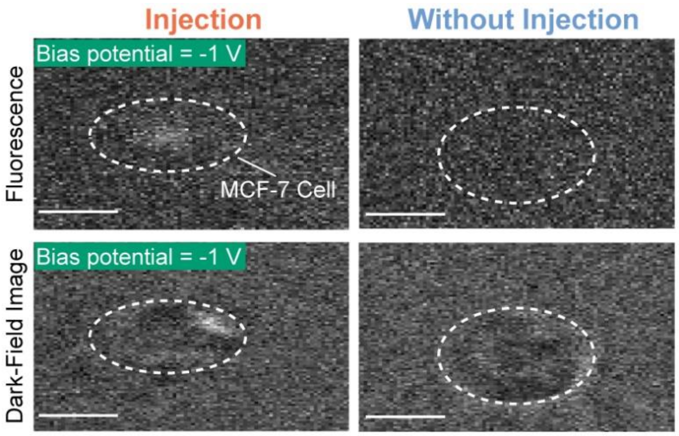

Supplementary Figure 13. Cell injection of 10 μM DMTPS-DCV using electrochemically nanopore manipulation. The fluorescence micrograph (upper) and dark-field image (bottom) of immobilized MCF-7 cell. Left: the DMTPS-DCV is injected into the cell, leading to the emission inside cell; Right: the controlled cell without AIEgen injection. Scale bars, 20 μm.

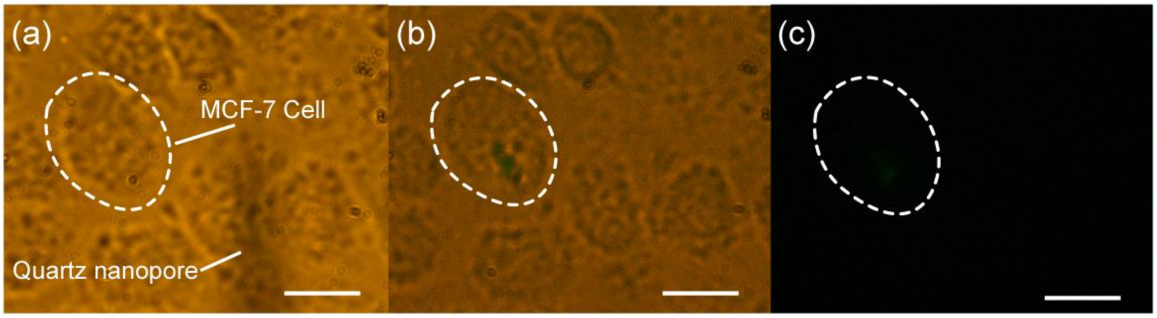

Supplementary Figure 14. Cell injection of 10 mM DMTPS-DCV using electrochemically nanopore manipulation. (a) The fluorescence micrograph of the immobilized MCF-7 cell before the injection. The shadow represents the quartz nanopore which is approaching to a single cell. The fluorescence micrograph (b) and dark-field image (c) of the immobilized MCF-7 of immobilized MCF-7 cell after the injection. The quartz nanopore has retreated from the cell. Scale bars, 20 μm.

Supplementary References

1. Mei, J. et al. Discriminatory detection of cysteine and homocysteine based on dialdehyde-functionalized aggregation-induced emission fluorophores. *Chem.-Eur. J.* **19**, 613-620 (2013).
2. Mei, J. et al. Siloles symmetrically substituted on their 2,5-positions with electron-accepting and donating moieties: facile synthesis, aggregation-enhanced emission, solvatochromism, and device application. *Chem. Sci.* **3**, 549-558 (2012).
3. Laforge, F. O., Carpino, J., Rotenberg, S. A. & Mirkin, M. V. Electrochemical attosyringe. *Proc. Natl. Acad. Sci. U.S.A.* **104**, 11895-11900 (2007).
