# Supplementary material for: Manipulating and visualizing the dynamic aggregation-induced emission within a confined quartz nanopore
Source: Nat Commun. 2018 Sep 7;9:3657. doi: 10.1038/s41467-018-05832-y (PMC6128826; doi:10.1038/s41467-018-05832-y)
Supplement: Supplementary file 3 — Description of Additional Supplementary Files [file 41467_2018_5832_MOESM3_ESM.pdf]

Description of Additional Supplementary Files

File Name: Supplementary Movie 1
Description: **Synchronized optical and electrical recording the reversible manipulating of DMTPS-DCV in a quartz nanopore.** The synchronized collection of electrical and optical signals for the reversible emission of DMTPS-DCV is guaranteed by the multichannel design of the analog to digital converter (ADC), then monitored by the computer.

File Name: Supplementary Movie 2
Description: **Reversible modulation of the AIEgens in a quartz nanopore.** The alternating applied bias potential between 1 V and -1 V inducing the reversible emission of DMTPS-DCV. The inside and outside nanopore filled with aqueous solution and 10 µM DMTPS-DCV acetonitrile solution, respectively. The 10 mM TBAPF6 and 10 mM KCl were used as organic and aqueous electrolyte to conduct the ionic flow.
